# Formal kinetics of H1N1 epidemic

**DOI:** 10.1186/1742-4682-6-23

**Published:** 2009-09-15

**Authors:** Konstantin G Gurevich

**Affiliations:** 1 UNESCO Chair in Healthy Life for Sustainable Development, Moscow State University of Medicine and Dentistry (MSDMU), Delegatskaya Street 20/1, 127473, Moscow, Russia

## Abstract

**Background:**

The formal kinetics of the H1N1 epidemic seems to take the form of an exponential curve. There is a good correlation between this theoretical model and epidemiological data on the number of H1N1-infected people. But this formal model leads to paradoxes about the dates when everyone becomes infected: in Mexico this will happen after one year, then in the rest of the world.

**Further implications of the formal model:**

The general limitations of this formal kinetics model are discussed. More detailed modeling is examined and the implications are examined in the light of currently available data. The evidence indicates that not more than 10% of the population is initially resistant to the H1N1 virus.

**Conclusion:**

We are probably only at the initial stage of development of the H1N1 epidemic. Increasing the number of H1N1-resistant people in future (e.g. due to vaccination) may influence the dynamics of epidemic development. At present, the development of the epidemic depends only on the number of people in the population who are initially resistant to the virus.

## Background

The first cases of swine influenza virus A (H1N1) were reported on 9 April 2009 in Mexico and 17 April 2009 in the USA [1]. For some experts, the H1N1 epidemic seems to be a potential global disaster [2]. Every 2-3 days, WHO publishes updated information [3] about the number of H1N1-infected people all over the world.

## Formal kinetics model

**Figure 1:**
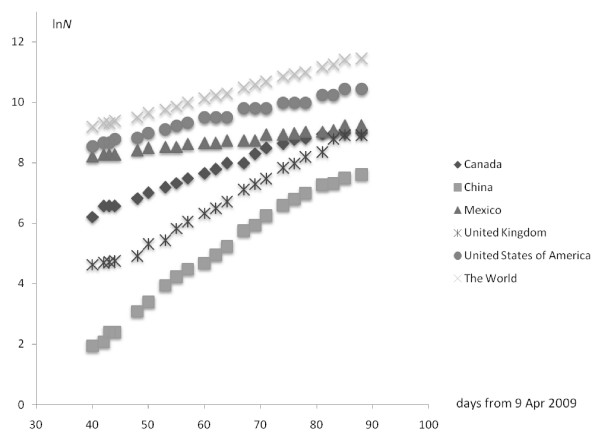
**The number of infected people in different countries**.

We took the number of H1N1-infected people in the countries showing the greatest spread from "zero time" (9 April) up to the most recent WHO update (6 Jul 2009). In half-logarithmic coordinates the number of infected people seems to be a linear function of time (Figure 1). From the formal kinetics point of view, this means that the number of H1N1-infected people can be described by the following function:

(1)

where *N *is number of infected people, *a, b *and *c *are constants, and *t *is time.

The parameters of formal model (1) were calculated and there was a very good correlation coefficient between the theoretical model and real epidemiological data for different countries and for the world (Table 1).

**Table 1:** Calculated values for model (1) for different countries

**Country**	**Number of citizens** [5]**, million people**	***a***	***b***	***c***	***t*_*last*_**	**Correlation coefficient between** **model and epidemiological data, %**
Canada	30	0.9249	0.06121	3.961	12.11.2009	95.3

China	1314.5	0.7458	0.1252	-2.8695	18.10.2009	95.1

Mexico	107	0.9948	0.02149	0.73488	14.07.2011	98.7

UK	53.4	0.9864	0.09821	0.4632	02.10.2009	98.2

USA	303.8	1.0075	0.03844	7.078	26.02.2010	99.1

The World	9756	0.9733	0.05028	7.1198	19.02.2010	99.7

Coefficient *b *is proportional to the velocity of the process:

(2)

i.e. the formal kinetics model of H1N1 epidemic development proposes that the velocity of the epidemic is directly proportional to the number of infected people. This implies that the H1N1 epidemic is only in its initial period and the number of H1N1-resistant people has not so far limited the epidemiological process.

Coefficient *b *seems to not to differ much among countries: its minimum is in the USA and its maximum in China, but the difference between minimum and maximum is only four-fold.

## Infection of the total population

From the formal kinetics model (1), the "last" time (*t*_*last*_), the time when all people in the country or in the world will be infected, can be easily calculated:

(3)

where *N*_0 _is the population size.

The calculated *t*_*last *_is given in Table 1. It characterizes the "pessimistic" variant of H1N1 epidemic development. According to the formal model, 19 Feb 2010 will be the day when all people in the world will be H1N1-infected. But in Mexico, all people will be infected only on 14 Jul 2011. This is the paradoxical result obtained from the formal kinetics model as it stands.

As a final comment we note that the formal model (2) does not take many relevant factors into account, i.e. active or passive immunity to H1N1 infection, different population densities in different countries and so on. In other words, this model has serious limitations as a practical approach.

## Avoiding the paradox

In a real situation in the human population we have at least three types of people: infected (*N*), sensitive (*M*) and resistant (*L*). In the simplest case, people become resistant after infection. The number of infected people increases proportionally because infected people make contact with sensitive people, and decreases proportionally owing to the number of infected people who become convalescent. The equations for such a model are well-known [4]:

(4)

where *b' *and *b" *are proportionality coefficients.

**Figure 2:**
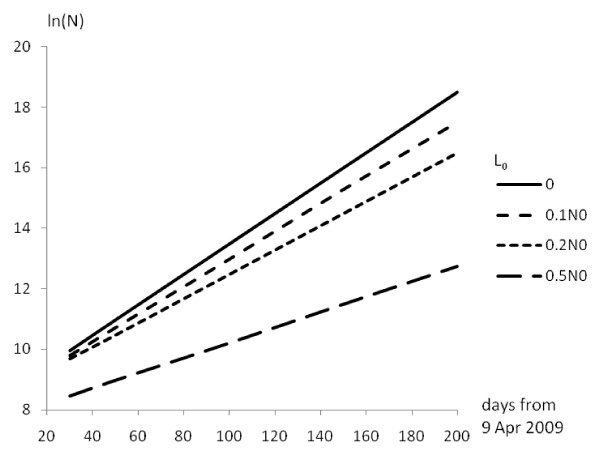
**Model (4) numerical solution for the World**. *b" *= *b'*/10.

For the initial period of epidemic development *M*>>*N*, i.e. *b'M-b"= b*. In this situation, system (4) reduces to equation (2). Also during the initial period, *M*<<*N*_0 _and *N*<<*N*_0_, so *b' *~ *b/N*_0_. In this limit, variations in *L*_0 _(initial number of resistant people) may yield a prognosis of the future development of the H1N1 epidemic (Figure 2). As can be seen from Figure 2, if *L*_0 _= 0.5*N*_0 _then the epidemic would develop from its outset differently from the way it has. If *L*_0 _= 0.2*N*_0_, the character of the epidemic must change after about 50 days of development (end of May 2009). If *L*_0 _= 0.1*N*_0_, the character of epidemic development will start to change from day 150 (beginning of Sept 2009). So, according to formal model (4), one can now suggest that the initial number of H1N1-resistant people is no more then 10% of the general population.

## Conclusion

Variation of *b" *(speed of immunity formation) in the interval from *b'*/1000 to *b'*/10 does not seriously influence the estimated parameters for model (4) with *L*_0 _= 0.1*N*_0_. As a result it may be suggested that we are now only at the initial stage of development of the H1N1 epidemic. It may also be suggested that increasing the number of H1N1-resistant people in future (e.g. due to vaccination) may influence the dynamics of epidemic development. But now the development of the epidemic depends only on the number of people in the population who are initially resistant to the H1N1 virus.

## Competing interests

The author declares that he has no competing interests.
